# Sample size calculation for a stepped wedge trial

**DOI:** 10.1186/s13063-015-0840-9

**Published:** 2015-08-17

**Authors:** Gianluca Baio, Andrew Copas, Gareth Ambler, James Hargreaves, Emma Beard, Rumana Z Omar

**Affiliations:** Department of Statistical Science, University College London, Gower Street, London, UK; MRC Clinical Trials Unit at University College London, CC, London, UK; Department of Social and Environmental Health Research, London School of Hygiene and Tropical Medicine, Keppel Street, London, UK; Department of Clinical, Educational and Health Psychology, University College London, Gower Street, London, UK; Department of Epidemiology and Public Health, University College London, Gower Street, London, UK

**Keywords:** Stepped wedge design, Sample size calculations, Simulation-based methods

## Abstract

**Background:**

Stepped wedge trials (SWTs) can be considered as a variant of a clustered randomised trial, although in many ways they embed additional complications from the point of view of statistical design and analysis. While the literature is rich for standard parallel or clustered randomised clinical trials (CRTs), it is much less so for SWTs. The specific features of SWTs need to be addressed properly in the sample size calculations to ensure valid estimates of the intervention effect.

**Methods:**

We critically review the available literature on analytical methods to perform sample size and power calculations in a SWT. In particular, we highlight the specific assumptions underlying currently used methods and comment on their validity and potential for extensions. Finally, we propose the use of simulation-based methods to overcome some of the limitations of analytical formulae. We performed a simulation exercise in which we compared simulation-based sample size computations with analytical methods and assessed the impact of varying the basic parameters to the resulting sample size/power, in the case of continuous and binary outcomes and assuming both cross-sectional data and the closed cohort design.

**Results:**

We compared the sample size requirements for a SWT in comparison to CRTs based on comparable number of measurements in each cluster. In line with the existing literature, we found that when the level of correlation within the clusters is relatively high (for example, greater than 0.1), the SWT requires a smaller number of clusters. For low values of the intracluster correlation, the two designs produce more similar requirements in terms of total number of clusters. We validated our simulation-based approach and compared the results of sample size calculations to analytical methods; the simulation-based procedures perform well, producing results that are extremely similar to the analytical methods. We found that usually the SWT is relatively insensitive to variations in the intracluster correlation, and that failure to account for a potential time effect will artificially and grossly overestimate the power of a study.

**Conclusions:**

We provide a framework for handling the sample size and power calculations of a SWT and suggest that simulation-based procedures may be more effective, especially in dealing with the specific features of the study at hand. In selected situations and depending on the level of intracluster correlation and the cluster size, SWTs may be more efficient than comparable CRTs. However, the decision about the design to be implemented will be based on a wide range of considerations, including the cost associated with the number of clusters, number of measurements and the trial duration.

## Background

Sample size calculations for a trial are typically based on analytical formulae [1], often relying on the assumption of (approximate) normality of some test statistic used for the analysis. In the case of cluster RCTs (CRTs), where clusters rather than individuals are randomised, the outcomes for participants within a cluster are likely to be more similar than those between clusters.

The most common approach to computing the optimal sample size for a CRT is to formally include some form of variance inflation, often expressed in terms of a design effect (DE) [2–7], the factor by which the sample size obtained for an individual RCT needs to be inflated to account for correlation in the outcome [8]. In the simplest case, the DE is computed as a function of the number of individuals in each cluster and the intracluster correlation (ICC), which quantifies the proportion of the total variance due to variation between the clusters. In practice, a preliminary size is computed as if the trial were an individual RCT and the sample size is obtained by multiplying this by the DE, which thus quantifies the inflation in the sample size resulting from the reduced amount of information due to the lack of independence across the observations. In the case of standard CRTs, there is a considerable literature dealing with more complicated scenarios, for example, when repeated measures are obtained from individuals within the clusters [9]. Stepped wedge trials (SWTs) are a variant of CRTs where all clusters receive the intervention in a randomised order. They also have additional features which need to be formally taken into account in the sample size calculations, including: the number of crossover points; the number of clusters switching intervention arm at each time point; possible time and/or lag effect, indicating that the intervention effect may not be instantaneous; and the dynamic aspects of the underlying population, for example, whether the data are collected for a SWT in a cross-sectional manner or they are repeated measurements on the same individuals.

The available literature for sample size and power calculations for a SWT is much less rich than that on parallel or cluster randomised trials. In addition to the risk of bias and logistic challenges [10, 11], this is perhaps one of the reasons for the limited development of trials based on the SWT design, at least until very recent times [11]. Indeed, many SWT studies published between 1950 and 2010 did not report formal sample size calculations, and for those which did, descriptions of the details were not adequate [12, 13]. Nonetheless, some improvements have been made over the last few years, and a number of papers have been published on sample size calculations for SWT. These include the pivotal paper published in 2007 by Hussey and Hughes (HH) [14], which provided both analytic formulae and the results of a simulation exercise for sample size calculations. Methods for the computation of DEs for a SWT have also been recently proposed [15, 16].

Despite the recent increase in the number of published trials using stepped wedge designs, a recent review on the reporting of the conduct of SWTs [11] suggests only a few studies mentioning the ICC and a justification for its assumed value, which effect sizes were adopted and the other assumptions on which the calculations were based. Of the 38 studies identified in the review, 8 did not report any form of sample size calculation (5 of these were only based on trial registration) and 10 used formulae for parallel or cluster RCTs. Of those accounting for the stepped wedge design, the most common method used was that of HH [14], while only one study used the DE defined by Woertman et al. [15], one used the method proposed by Moulton et al. [16] and three used simulations to compute the sample size. Of the 30 studies which reported a sample size calculation, just 19 included the ICC, of which only a few appeared to be based on previous research. Given the often longitudinal nature of SWTs, it is surprising that only 9 accounted for possible drop-out. Moreover, the sample size calculations did not always match the methods of analysis undertaken, and although many of the studies used repeated measures designs, adjusting for covariates and assessing possible time by intervention interactions effects, they did not take these into account in the sample size calculations.

Existing guidance on sample size calculations for a SWT is also limited by the fact that it has mainly focussed solely on cross-sectional designs, ignoring the more complex clustering which occurs in studies where repeated measurements are taken from the same individuals [14–16]. For cross-sectional outcome data, these are assumed to be measured at discrete times linked to the timing of the ‘steps’ (crossover points) in the design and it is assumed that the analysis will include data from one crossover after all clusters have changed to the intervention condition and from one crossover before. Other typical assumptions include equal cluster sizes, no intervention by time interactions, no cluster-by-intervention effect and categorical time effects (we return to this point later).

Very recently, Hemming et al. [17] have provided analytical formulae for power calculations for specific variations on HH’s basic formulation. These include the case of multiple levels of clustering, for example, an intervention being implemented in wards within hospitals, and what they term the ’incomplete’ SWT design, in which clusters may not contribute data for some time periods, for example, because of implementation periods in which the clusters transition from the control to the intervention arm, or to avoid excessive measurement burden. Nevertheless, as suggested in [18], to date reliable sample size algorithms for more complex designs, such as those using cohorts rather than cross-sectional data, have not yet been established.

The objective of this paper is to provide a critical review of the analytical methods currently available for sample size calculations for a SWT and to suggest the potential extension of these closed-form methods to simulation-based procedures, which may be more appropriate and offer more flexibility in matching the complexity of the model used for the analysis. We show the results of a simulation study, comparing the performance of the simulation-based approach to that of the closed-form calculations, and finally give some recommendations on when either procedure may be more accurate.

## Methods

### Analytical methods for sample size calculations in a stepped wedge trial

Before we proceed, we note that since this is a methodological paper, no ethical approval was required for any of the aspects we present and discuss in the following sections. There are three main papers detailing the sample size requirements for a SWT. The first one is that of HH, who proposed power calculations for stepped wedge designs with cross-sectional data and investigated the effect on power of varying several parameters. The basic model considered by HH assumes *I* clusters, *J* crossover points and *K* individuals sampled per cluster at each time point. In the most basic formulation, the observed continuous response is then modelled as *Y*_*ijk*_=*μ*_*ij*_+*e*_*ijk*_, where

$$\mu_{ij} = \mu+ \alpha_{i} + \beta_{j} + X_{ij}\theta $$

is the cluster- and time-specific mean, while $e_{\textit {ijk}}\sim \text {Normal}(0,{\sigma ^{2}_{e}})$ represent independent individual-level error terms (within-cluster variability). Here, *μ* is the overall intercept, $\alpha _{i} \sim \text {Normal}(0,\sigma ^{2}_{\alpha })$ are a set of cluster-specific random effects, *β*_*j*_ are fixed effects for time *j*, *X*_*ij*_ is an intervention indicator taking on the value 1 if cluster *i* is given the active intervention at time *j* and 0 otherwise, and *θ* is the intervention effect. This model implies that the response *Y*_*ijk*_ is normally distributed with mean *μ*_*ij*_ and total variance ${\sigma ^{2}_{y}}=\sigma ^{2}_{\alpha }+{\sigma ^{2}_{e}}$, while the cluster-level variance is $\frac {\sigma ^{2}_{\alpha }+{\sigma ^{2}_{e}}}{K}\left [1+(K-1)\rho \right ]$, where $\rho =\frac {\sigma ^{2}_{\alpha }}{\sigma ^{2}_{\alpha }+{\sigma ^{2}_{e}}}$ is the ICC.

HH’s power calculations are based on the Wald test statistic, computed as the ratio between the point estimate of the intervention effect and its standard deviation. The main complexity lies in the computation of the variance of the estimator of the intervention effect; nevertheless, in the relatively standard case considered by HH, this can be expressed analytically as

$$V(\theta) =\frac{I\sigma^{2}(\sigma^{2}+J\sigma^{2}_{\alpha})}{(IU-W)\sigma^{2}+(U^{2}+IJU-JW-IV)\sigma^{2}_{\alpha}}, $$

where $\sigma ^{2}=\frac {{\sigma ^{2}_{e}}}{K}$, while $U=\sum _{\textit {ij}}X_{\textit {ij}}$, $W=\sum _{j}\left (\sum _{i} X_{\textit {ij}}\right)^{2}$ and $V= \sum _{i}\left (\sum _{j} X_{\textit {ij}}\right)^{2}$ are all easily computable functions of the design matrix. The within- and between-cluster variations are usually not known a priori, but similar to the case of standard parallel or cluster RCTs, suitable estimates can be plugged in, perhaps using information from previous or pilot studies.

The power is computed as

$$\text{Power}=\Phi\left(\frac{\theta}{\sqrt{V(\theta)}}-z_{\alpha/2}\right) $$

where *Φ* is the cumulative standard normal distribution and *z*_*α*/2_ is its (1−*α*/2)−th quantile. This formulation assumes exchangeability across time within each cluster; that is, the same correlation is assumed between individuals regardless of whether or not they are exposed to the intervention or the control. Furthermore, the model takes into account external time trends, but assumes they are equal for all clusters. Incorporating such time effects is necessary for SWTs, particularly for cases where the outcome is likely to vary over time [19].

Drawing on asymptotic theory, HH’s calculations can be easily extended to the case in which the outcome is not normally distributed. Using HH’s calculations, Hemming and Girling [20] have also written a Stata [21] routine steppedwedge, which allows continuous, binary and rate outcomes. The routine allows the specification of the number of clusters randomised at each crossover, the number of crossover points and the average cluster size.

### Analytical sample size calculations based on design effects

As an alternative to HH’s formulation, some authors have proposed sample size calculations based on the derivation of a design effect, an approach commonly used in standard parallel CRTs. For example, Woertman et al. [15] suggest the use of (what they term) a DE, based on HH’s formulation. Their approach assumes that the outcome measurements are obtained from each cluster at a number of discrete time points and that the number of participants measured at each of these crossover points is the same across times and clusters. The formula to compute the correction factor (CF) depends on the number of crossover points at which the clusters switch to the intervention (*J*), the number of baseline measurement times (*B*), the number of measurement times during each crossover (*T*), the number of participants measured at each time in each cluster (*K*) and the ICC *ρ*:

$$\text{CF} = \frac{1+\rho(JTK+BK-1)}{1+\rho\left(\frac{1}{2}JTK+BK-1\right)}\frac{3(1-\rho)}{2T\left(J-\frac{1}{J}\right)}. $$

The overall sample size in terms of participants (each contributing one measurement) is then obtained as

$$n = n_{RCT}\times (B+JT)\times \text{CF} $$

where *n*_*RCT*_ is the sample size computed for a corresponding parallel individual RCT without baseline data. Thus, we note here that the correction factor cannot be considered as a DE in a conventional sense, and in fact the proper formulation is

$$\text{DE}_{W} = (B+JT)\times \text{CF}. $$

The underlying assumptions behind this formulation are similar to those used by HH, with the exceptions that the same number of clusters switches at each crossover and the number of measurements after each crossover is constant. Because the calculation of this DE is based on HH’s model, it applies only to cross-sectional settings, so that each measurement is from a different individual participant. For example, measurements may arise from sampling a small fraction of a large cohort at each time point, or repeated cohorts of new individuals may be exposed to intervention or control conditions at each crossover and provide outcome measures at the end of the crossover. However, Woertman et al. erroneously applied their DE to a setup in which the same cohort of individuals was observed repeatedly over time.

Often, in a SWT measurements are not obtained at discrete times; for example, consider the commonly conducted design termed a *continuous recruitment short period exposure* design, in [22]. In such a design DE_*W*_ can be used by considering the cluster size *K* to be the number of individuals recruited (that is, providing outcome measurements) per cluster during each crossover, setting *T*=1 and *B* equal to the ratio of the number of outcome measurements obtained before roll-out to the number obtained during each subsequent crossover.

A similar methodology based on the computation of a specific DE for a SWT was proposed by Moulton et al. [16], specifically for survival data. Their DE considers the case where the main analysis consists of comparisons of the outcome for the clusters receiving the intervention to those who have yet to receive it. Assuming that all the clusters receive the intervention by the last time point *J*, in this case the test is based on a log-rank statistic

$$Z=\frac{\sum_{j=1}^{J} \left[{d_{j}^{1}} -{Y_{j}^{1}}\left(\frac{d_{j}^{*}}{Y_{j}^{*}} \right)\right]}{ \sqrt{\sum_{j=1}^{J} \frac{{Y_{j}^{1}}}{Y_{j}^{*}} \left(1- \frac{{Y_{j}^{1}}}{Y_{j}^{*}} \right) \left(\frac{Y_{j}^{*}-d_{j}^{*}}{Y_{j}^{*}-1} \right)d_{j}^{*}}} $$

where: $\{{d_{j}^{0}},{d_{j}^{1}}\}$ indicate the number of new cases at time *j*, respectively in the clusters that are not treated (labelled by the superscript 0) and in those that are treated (labelled by the superscript 1); $\{{Y_{j}^{0}},{Y_{j}^{1}}\}$ indicate the number of subjects at risk at time *j* in the untreated and treated clusters, respectively; $d_{j}^{*}={d_{j}^{0}}+{d_{j}^{1}}$ and $Y_{j}^{*}= {Y_{j}^{0}}+{Y_{j}^{1}}$ are the total incident cases and number at risk at time *j*.

The log-rank statistic can be computed assuming either a standard CRT scheme or a time-varying allocation of the clusters to the intervention. The comparison between its values under the two scenarios provides a measure of the DE for a SWT. The final sample size calculation is then performed by inflating a suitable standard sample size (based on [23]) by this factor. In the original paper [16], the computation of the values for ${d_{j}^{0}}$ and ${d_{j}^{1}}$ is based on simulations, but we note here that their procedure is fundamentally different from the one we describe in the next sections and, as such, we still class this method as a form of analytical calculation.

### Limitations of analytical sample size calculations

As mentioned above, the main limitation of the analytical methods of [14–16] is that they are not directly applicable when repeated measures are taken on the same individuals over time, due to the additional level of correlation implied in this case. Thus, calculations based on cross-sectional data are likely to overestimate the required sample size for a design involving repeated measurements.

More importantly, while analytical formulae and DEs are generally simple to use, the extra complexity of several potential SWT designs means that these cannot be directly used without applying the necessary modifications to the original formulation, to align the design and analysis models for the SWT under consideration. Consequently, the use of simulation-based methods has been suggested as a valid and more general alternative [24], which can be used to cater for the specific features of a SWT.

### Simulation-based sample size calculations

The use of a simulation-based approach to determine the optimal sample size for a study is not a new concept, nor is it specific to the design of SWTs [25–27]. Stated briefly, the idea is to consider a model to represent the data generating process (DGP), which describes how the researchers envisage the way in which the trial data will eventually be observed. This should be the model that is used to analyse the data, after the study has been conducted. Using the assumed DGP, data can be simulated a large number of times and the resulting ’virtual trials’ can be analysed using the proposed analysis model.

Some of the parameters may be varied across the simulations: for example, it is interesting to investigate the results obtained by varying the total number of observations. The optimal sample size is set to the minimum number of subjects for which the proportion of simulated trials that correctly deem the intervention as significant at the set *α*−level is greater than or equal to the required power.

The main advantage of using simulation-based approaches to determine the sample size is that, in principle, any DGP can be assumed, no matter how complex. Of course, trials associated with more complicated designs will also require longer computational time to produce a sufficient number of runs to fully quantify the operating characteristics, for example, in terms of the relationship between power and sample size. This is essential to estimate the required sample size properly.

#### Cross-sectional data designs

The simplest situation is probably that of a repeated cross-sectional design in which measurements are obtained at discrete times from different individuals. This manner of taking measurements is consistent with an *open cohort* SWT in which a small fraction of the participants in each trial cluster is sampled for measurements at each time [22].

In this case, the general framework for the simulation-based approach can be described as follows. Individual variability in the observed data *Y*_*ijk*_ is described using a suitable distribution depending on the nature of the outcome and characterised by cluster- and time-specific mean *μ*_*ij*_ and an individual (within-cluster) level variance ${\sigma ^{2}_{e}}$. The mean of the outcome is described by a linear predictor, on a suitable scale:

$$\phi_{ij}=g(\mu_{ij}) = \mu+\alpha_{i}+\beta_{j}+X_{ij}\theta. $$

When considering symmetrical and continuous data, we may assume a normal distribution, and thus the function *g*(·) is just the identity. For example, [28] assessed the impact of a nutritional intervention on preventing weight loss using this formulation. The assumption of normality is by no means essential: for instance, if we were aware of potential outliers, we could assume a more robust *t* distribution for the observed data.

In a simulation-based framework, it is straightforward to extend this structure to account for other types of outcomes; for example, binary responses are appropriately dealt with by assuming a Bernoulli distribution for the individual data and then considering a log-linear predictor on the odds, that is, *g*(*μ*_*ij*_)=logit (*μ*_*ij*_). This is the framework used by [29] to identify the proportion of patients obtaining a pre-specified weight loss, that is, modifying the definition of the primary outcome for the trial of [28].

Similarly, it is possible to consider count data modelled assuming a Poisson distribution and then a log-linear predictor for the mean *g*(*μ*_*ij*_)= log (*μ*_*ij*_), as in the trial described by Bacchieri et al. [30], who assessed the effectiveness of a cycling safety program by determining the number of accidents over time pre- and post-intervention. Notice also that this definition of the linear predictor applies to continuous and skewed observations, which can be modelled using a lognormal or a gamma distribution.

#### Closed cohort designs

Another relevant situation is represented by repeated measurements on the same cohort of individuals, termed a *closed cohort* in [22]. Under this design, it is necessary to account for the induced correlation between the measurements obtained by the same individual. This is easily done by adding a random effect $v_{\textit {ik}}\sim \text {Normal}\,(0,{\sigma _{v}^{2}})$, which is specific to the *k*-th individual in cluster *i*, to each of the linear predictors described above. In the most basic formulation this then becomes

$$\phi_{ij}=g(\mu_{ij}) = \mu+\alpha_{i}+\beta_{j}+X_{ij}\theta + v_{ik}, $$

but of course it is possible to extend this to combine the cluster- and individual-specific random effect with other features. This construction can be easily extended to account for ’multiple layers of clustering’ (similar to those mentioned in [17]).

#### Modelling extensions for more complex data generating processes

The use of simulation-based sample size calculations proves particularly effective to model the extra complexity implied by non-standard cases. Examples are the inclusion of additional covariates, which may or may not depend on time or the cluster allocation to the intervention, as well as more structured effects (such as interactions or higher order effects for the intervention or other covariates included in the model, such as quadratic trends).

One relevant potential extension to the model is to consider a data generating process including an additional cluster-specific random effect, so that the linear predictor becomes

$$\phi_{ij}=g(\mu_{ij}) = \mu+\alpha_{i}+\beta_{j}+X_{ij}(\theta +u_{i}), $$

depending on the suitable link function *g*(·). Here $u_{i}\sim \text {Normal}\,(0,{\sigma _{u}^{2}})$ and ${\sigma _{u}^{2}}$ is a variance term common to all the clusters. These terms can be interpreted as cluster-specific variations in the intervention effect. Alternatively, the term (*θ*+*u*_*i*_) can be interpreted as a cluster-varying slope for the intervention effect.

This structure may be relevant, for example, to address cases where variations in how the intervention is implemented in different clusters are likely to occur. Notice that the data will inform the estimation of ${\sigma _{u}^{2}}$ so that, if there is no evidence of cluster-specific variations in the intervention effect, this parameter will be estimated to be 0 and thus all clusters will be estimated to have the same intervention effect. In practical terms, in order to perform the simulation-based sample size calculations, it is necessary to provide an estimate of the variance ${\sigma _{u}^{2}}$. This may not be known with precision, and thus it is helpful to perform sensitivity analysis on the actual choice.

Another interesting extension to the framework involves including a random effect to model time, for example $\beta _{j} \sim \text {Normal}\,(0,\sigma _{\beta }^{2})$ with $\sigma ^{2}_{\beta }$ specifying a variance term common to all time points. Alternatively, the time effect may be specified using more complex specifications such as random walks. HH have already discussed this possibility and suggested that it “*might be particularly appropriate if temporal variations in the outcome were thought to be due to factors unrelated to changes in the underlying disease prevalence (e.g. changes in personnel doing outcome surveys)*”. Again, this would not have any substantial implication on our simulation methods, although the additional time-specific random effect would make the structure of the models more complex and thus potentially increase the computational time.

Notice that these more general constructions involve the specification of suitable values for additional parameters and that, while often providing a more robust option, as seems intuitively obvious, these complications in the modelling structure will generally increase the required sample size. In addition, these more complex models apply equally to cross-sectional and cohort designs.

#### Simulation procedure

Regardless of the modelling assumptions for the outcomes or the form assumed for the cluster- and time-specific mean, the simulation procedure can be schematically described as follows.

- Select a total sample size *n* (for example, total number of individuals measured) and a suitable combination of the number of clusters *I* and time points *J*.
- Provide an estimate of the main parameters. These can be derived from the relevant literature or expert opinion. We recommend thorough sensitivity analyses to investigate the impact of these assumptions on the final results, in terms of optimal sample size. In the simplest case described above, these include:
  - The design matrix ***X***, describing how the clusters are sequentially allocated to the intervention arm;
  - The intercept *μ*, which represents an appropriate baseline value;
  - The assumed intervention effect *θ*;
  - The between- and within-cluster variances $\sigma ^{2}_{\alpha }$ and ${\sigma ^{2}_{e}}$. Given the relationship between these two variances and the ICC, it is possible to supply one of them and the ICC, instead.
- Simulate a dataset of size *n* from the assumed model. In the simplest case mentioned above, this amounts to the following steps:
  - Simulate a value for each of the random cluster-specific effects $\alpha _{i} \sim \text {Normal}(0,\sigma ^{2}_{\alpha })$;
  - Simulate a value for the fixed time-specific effect *β*_*j*_, for example, a linear trend;
  - Compute the linear predictor by plugging in the values for the relevant quantities; note that this represents the mean of the outcome, on a suitable scale;
  - Simulate a value for the outcome from the assumed distribution and using the parameters derived in the previous steps.
- Analyse the resulting dataset and record whether the intervention effect is detected as statistically significant.

Steps *iii* and *iv* are repeated for a large number *S* of times for each of the selected values of *n*, and the proportion of times in which the analysis correctly detects the assumed intervention effects as significant is used as the estimated power. The lowest value of *n* in correspondence of which the estimated power is not less than the pre-specified threshold (usually 0.8 or 0.9) is selected as the optimal sample size. A Monte Carlo estimate of the error around the estimated power can be easily computed and used as a guideline to determine the optimal number of simulations to be used. In many situations, a value of *S* in the order of 1,000s will suffice.

Sensitivity to the choice of the fundamental parameters can be checked by selecting different values and repeating the procedure. For example, it is possible to assess the impact of varying the cluster size. An alternative version of this algorithm may involve the adoption of a fully Bayesian approach [31]; this amounts to modelling the uncertainty in the basic parameters using suitable probability distributions. For example, one could assume that, based on currently available evidence, the between-cluster standard deviation is likely to lie in a range between two extreme values *a* and *b*. This may be translated, for example, into a prior uniform distribution defined in (*a*,*b*). The sample size calculations would then account for the extra uncertainty in the actual value of this parameter. The benefits of this strategy are of course higher if genuine information is available to the researchers.

## Results

We used both analytical and simulation-based calculations to assess several aspects of a SWT, in terms of sample size calculations.

As suggested by Hemming et al. [32], in some cases the information provided by the within-cluster analysis in a SWT may lead to an improvement in efficiency, in comparison to a CRT with the same number of overall measurements. This is due to the fact that not only are within-cluster comparisons used to estimate intervention effects, but also within-subject comparisons [33]. Thus, we first assess the efficiency of a SWT against a standard CRT by comparing the sample size resulting from applying several alternative calculation methods and upon varying the ICC.

Then, we validate the simulation-based approach against the analytical formulation of HH, for cross-sectional data. Finally, we use the simulation-based approach to assess the impact of varying the basic parameters to the resulting sample size/power, in the case of continuous and binary outcomes and assuming both cross-sectional data and the closed cohort design.

All simulations and analyses were performed using the freely available software R [34]. A package will be made available, containing suitable functions to perform analytic and simulation-based calculations to determine the sample size of a SWT.

### SWT versus CRT

For all types of outcomes described above and assuming cross-sectional data, we computed the number of clusters required to obtain 80 % power to detect a specified intervention effect using the following methods: a standard inflation factor based on a CRT (results are presented in the first two columns of Table 1); the DE of Woertman et al. (the third column); the analytical values of HH (the fourth column).

**Table 1:** Estimated number of clusters for three sample size calculation methods used in SWTs, as a function of the ICC and outcome type (continuous, binary and rate) to obtain 80 % power

ICC	Standard CRT inflation factor		DE inflationfactor based onWoertman et al.	Analytical power based on HH
	*K*=20,*J*=1	*K*=120,*J*=1	*K*=20,*J*=6	*K*=20,*J*=6
Continuous outcome^*a*^				
0	26	5	8	9
0.1	74	55	12	12
0.2	122	105	11	11
0.3	170	155	10	10
0.4	218	205	9	9
0.5	266	256	7	7
Binary outcome^*b*^				
0	25	5	8	10
0.1	71	53	12	13
0.2	117	101	11	12
0.3	163	149	9	11
0.4	209	197	8	10
0.5	256	246	7	8
Count outcome^*c*^				
0	24	4	8	8
0.1	69	51	11	11
0.2	114	98	10	10
0.3	159	145	9	9
0.4	203	192	8	8
0.5	248	238	7	

^*a*^Intervention effect = −0.3785; *σ*_*y*_=1.55; sample size for a parallel RCT = 253 subjects per arm.

^*b*^Baseline outcome probability = 0.26; OR = 0.56; sample size for a parallel RCT = 243 subjects per arm.

^*c*^Baseline outcome rate = 1.5; RR = 0.8; sample size for a parallel RCT = 236 subjects per arm.

Notation: *K*= number of subjects per cluster; *J*= total number of time points, including one baseline

For all the outcomes, we considered a linear time trend and arbitrarily assumed a standardised effect size of around 0.25, obtained by setting the following inputs:

- *Continuous outcome*: baseline value *μ*=0.3; intervention effect *θ*=−0.3785; total standard deviation *σ*_*y*_=1.55.
- *Binary outcome*: baseline probability *μ*=0.26; intervention effect OR = exp(*θ*)=0.56.
- *Count outcome*: baseline rate *μ*=1.5; intervention effect RR = exp(*θ*)=0.8.

The values selected for the examples are loosely based on three of the trials we have reviewed [28–30].

For the two DE methods, we first computed the sample size required for a parallel RCT and then applied the suitable inflation factor. In the SWT design, we considered a common setting with *K*=20 subjects per cluster at each of a total of *J*=6 time points at which measurements were collected, that is, one baseline time at which all the clusters are in the control arm and 5 times at which the clusters sequentially switch to the intervention arm. Conversely, we considered two cases for the CRT: in the first one, we assumed the same number of measurements per cluster as in the SWT *K*=20, while in the second we assumed a cluster size equal to the total number of subjects in the corresponding SWTs (that is, 120 subjects, each measured at one single time point). We programmed the analytical calculations of HH in R and validated the output using the steppedwedge routine in Stata.

For all outcomes, we varied the ICC from 0, indicating no within-cluster correlation, to 0.5, which can be considered a high level of correlation, particularly in clinical settings. The methods discussed here are all based on the assumption that information is provided in terms of the total variance ${\sigma _{y}^{2}}$, which is in turn used to determine the between-cluster variance $\sigma _{\alpha }^{2}={\sigma _{y}^{2}}\rho $. This poses no problem in the computation of DE_*W*_ and the HH method, since they are both based on (approximate) normality of the outcomes. Thus, it is easy to control which source of variation is inputted through the variance parameter, which is separate from the linear predictor.

Table 1 shows that, in comparison with the standard CRT, the SWT can be much more efficient, under the settings we have considered. As previously reported [14], for increasingly larger values of the ICC (roughly speaking, greater than 0.1), the total number of measurements computed as *I*(*J*+1)*K* required to achieve 80 % power is increasingly smaller for a SWT than for either form of the CRT that we consider here. On the contrary, for very small values of the ICC, the two CRTs considered in Table 1 require a marginally smaller number of observations. This result is consistent across the three types of outcomes.

The DE computed using the method of Woertman et al. produces results very similar to those of the original HH calculations, particularly for continuous and count outcomes, in which cases the computed number of clusters is identical for the two methods.

### Simulation-based versus analytical sample size calculations

We then compared the results of the simulation-based approach applied to three types of outcomes with the HH analytical calculations. Notice that in the binary and count outcome cases it is more cumbersome to assume that information is provided in terms of the total variance. This is because, unlike the normal distribution, the Bernoulli and Poisson distributions are characterised by a single parameter, which simultaneously determines both the linear predictor and the variance. Consequently, because the linear predictor includes the cluster-specific random effects *α*_*i*_, assuming a fixed total variance ${\sigma ^{2}_{y}}$ implies a re-scaling of the baseline value *μ* to guarantee that the resulting total variance approximates the required value.

For this reason, when using a simulation-based approach for non-normally distributed outcomes it is easier to provide information on the within-cluster variance ${\sigma ^{2}_{e}}$ as input, which is then used to determine the between-cluster variance as $\sigma ^{2}_{\alpha }={\sigma ^{2}_{e}}\frac {\rho }{1-\rho }$. Since it is also possible to provide the within-cluster variance as input for the HH calculations, we use this strategy here, while keeping the numerical values from the previous example. This explains why the numbers for the method of HH in Table 2 differ from those in Table 1.

**Table 2:** Comparison of the simulation-based approach with the analytical formulae of HH. The cells in the table are the estimated number of clusters as a function of the ICC and outcome type (continuous, binary and rate) to obtain 80 % power

ICC	Analytical power based on HH	Simulation-based calculations
	*K*=20,*J*=6	*K*=20,*J*=6
Continuous outcome^*a*^		
0	9	9
0.1	13	13
0.2	14	13
0.3	14	14
0.4	14	14
0.5	14	14
Binary outcome^*b*^		
0	11	15
0.1	17	16
0.2	18	17
0.3	18	18
0.4	18	18
0.5	18	18
Count outcome^*c*^		
0	8	8
0.1	13	12
0.2	13	12
0.3	13	12
0.4	13	11
0.5	13	11

^*a*^Intervention effect = −0.3785; *σ*_*e*_=1.55.

^*b*^Baseline outcome probability = 0.26; OR = 0.56.

^*c*^Baseline outcome rate = 1.5; RR = 0.8.

Notation: *K*= number of subjects per cluster; *J*= total number of time points, including one baseline

The simulation-based power calculations are obtained by using the procedure described in the previous sections, repeating the process 1 000 times and assessing the resulting power within Monte Carlo error. As shown in Table 2, there was very good agreement between the method of HH and our simulations, particularly for the case of continuous outcome in which the results were identical. For binary and count outcome, the estimated numbers of clusters required to obtain 80 % power were slightly less aligned between the simulations and the method of HH. This is not entirely surprising, given that HH assume approximate normality, while our simulations directly address non-normality using binomial and Poisson models, respectively.

### Closed cohort design versus cross-sectional data: continuous and binary outcomes

#### Effect size and ICC

**Fig. 1:**
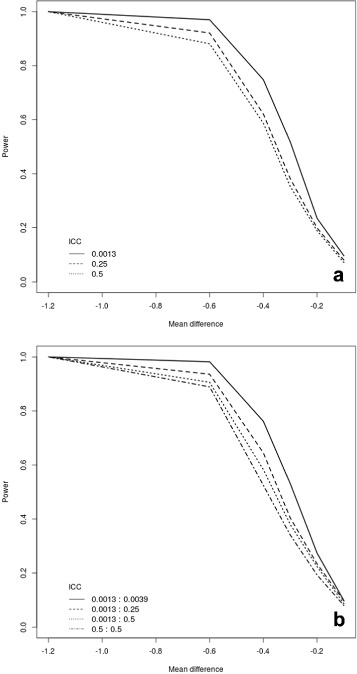
Power curves for a continuous outcome assuming: 25 clusters, each with 20 subjects; 6 time points including one baseline. We varied the intervention effect size and the ICC variations. Panel (**a**) shows the analysis for a repeated closed cohort (cross-sectional) design, while panel (**b**) depicts the results for a closed cohort design. In panel (**b**) the selected ICCs are reported for cluster and participant level

**Fig. 2:**
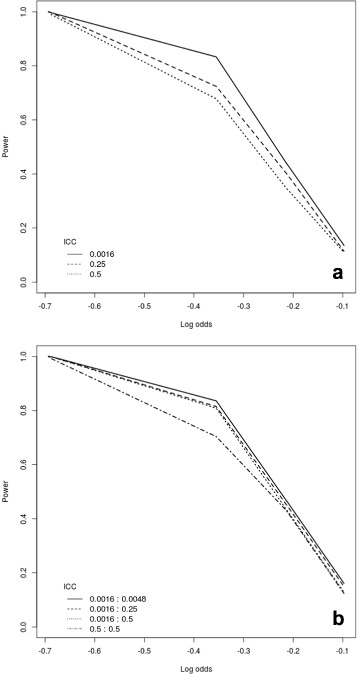
Power curves for a binary outcome assuming: 25 clusters, each with 20 subjects; 6 time points including one baseline. We varied the intervention effect size and the ICC variations. Panel (**a**) shows the analysis for a repeated closed cohort (cross-sectional) design, while panel (**b**) depicts the results for a closed cohort design. In panel (**b**) the selected ICCs are reported for cluster and participant level

Figures 1 and 2 show the power computed using our simulation-based approach as a function of the assumed effect size and the ICC for the continuous and binary outcome, respectively. We assume *I*=25 clusters each with *K*=20 subjects and a total of *J*=6 measurements. In both figures, panel (a) shows the results for the cross-sectional data, while panel (b) depicts those for the closed cohort design.

It is clear that large increases in the ICC at the cluster level for cross-sectional data result in a decline in power. In the closed cohort design case, we assessed the sensitivity of different specifications of the ICC both at the cluster and at the participant level. While in the case of continuous outcomes, changes in the ICC seem to only marginally affect the power, when considering a binary outcome, large values of the ICC (particularly at the cluster level) seem to reduce the power more substantially. In any case, the impact of the ICC appears less important than that of the mean difference.

#### Number of crossover points

**Fig. 3:**
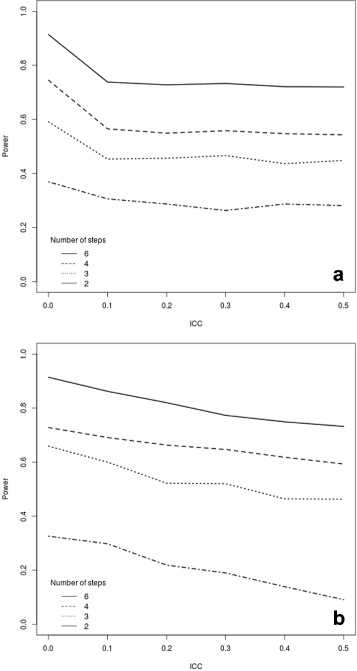
Power curves for a continuous outcome assuming 24 clusters, each with 20 subjects. We varied the ICC and the number of randomisation crossover points. Panel (**a**) shows the analysis for a repeated closed cohort (cross-sectional) design, while panel (**b**) depicts the results for a closed cohort design (assuming individual-level ICC of 0.0016)

**Fig. 4:**
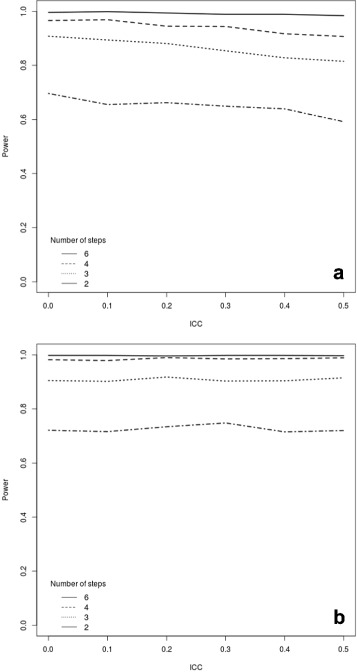
Power curves for a binary outcome assuming 24 clusters, each with 20 subjects. We varied the ICC and the number of randomisation crossover points. Panel (**a**) shows the analysis for a repeated closed cohort (cross-sectional) design, while panel (**b**) depicts the results for a closed cohort design (assuming individual-level ICC of 0.0016)

Figures 3 and 4 illustrate the effect of varying the number of clusters randomised each time and the number of crossover points with continuous and binary outcomes, respectively.

We assumed a fixed setup including *I*=24 clusters and varied the total number of crossover points *J* from 6 (that is, 4 clusters randomised at each time) to 2 (that is, 12 clusters randomised at each time). In both designs, we assume that subjects are measured once at each time point and that there is an individual level ICC of 0.0016 (again loosely based on the setting presented in [28, 29]). Therefore, for cross-sectional data we assume more individuals are measured per cluster with a larger number of crossover points, and for a closed cohort setting, we assume more measurements are taken on each individual with a larger number of crossover points.

Not surprisingly, the highest power is consistently observed as the number of crossover points increases and thus the number of clusters randomised at each crossover decreases. Consequently, optimal power will be achieved when only one cluster switches to the intervention arm at each time point. However, as noted previously by HH, in some practical cases it may be unfeasible for logistic reasons to have a large number of crossover points. Thus, measurement points should be maximised within the constraints of resource availability. In line with [35], the power gains from increasing the number of crossover points are not linear — with smaller gains when moving from four to six than when going from two to three crossover points. Given the potential additional cost of increasing the number of crossover points and resulting total number of measurements, it may not pay off to inflate the number of crossover points substantially.

#### Time effect

**Fig. 5:**
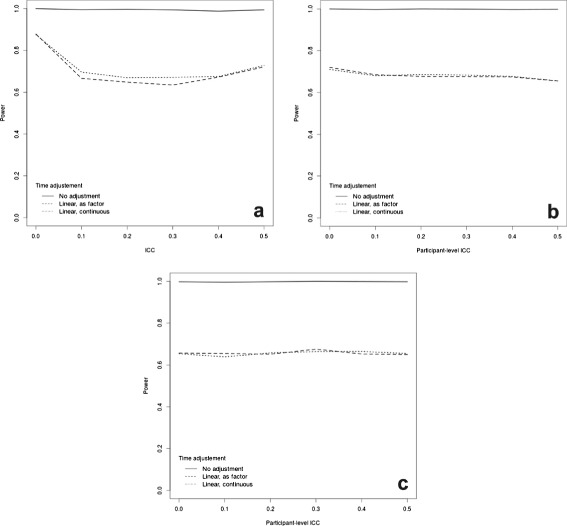
Power curves for a continuous outcome assuming 25 clusters, each with 20 subjects and 6 time points at which measurements are taken (including one baseline time). We varied the way in which the assumed linear time effect is included in the model (if at all). Panel (**a**) shows the results for a repeated cohort design; panel (**b**) shows the results for the closed cohort design, assuming a cluster-level ICC of 0.1 and varying the participant-level ICC; panel (**c**) shows the results for the closed cohort design, assuming a cluster-level ICC of 0.5 and varying the participant-level ICC

Failure to include a time effect in the analysis model, when one was assumed in the DGP, significantly but erroneously inflated the power. Figure 5 shows our analysis for a continuous outcome, assuming *I*=25 clusters, each with *K*=20 subjects and a total of *J*=6 measurements; panel (a) describes the case of a repeated cohort design, while panels (b) and (c) consider the case of a cohort design with individual level ICC of 0.1 and 0.5, respectively.

For the repeated cohort design, the power was also slightly inflated when time was included in the model as a continuous as opposed to a factor variable. The greater impact of variations in low ICC values for the repeated cohort design is clearly visible, as is the lesser sensitivity of the closed cohort design to variations in the within-cluster correlation. Studies based on continuous outcomes would therefore benefit from the use of a closed cohort design when there is substantial uncertainty on the ICC at the cluster level; however, there does not appear to be a general benefit of repeated measures over cross-sectional measurements.

**Fig. 6:**
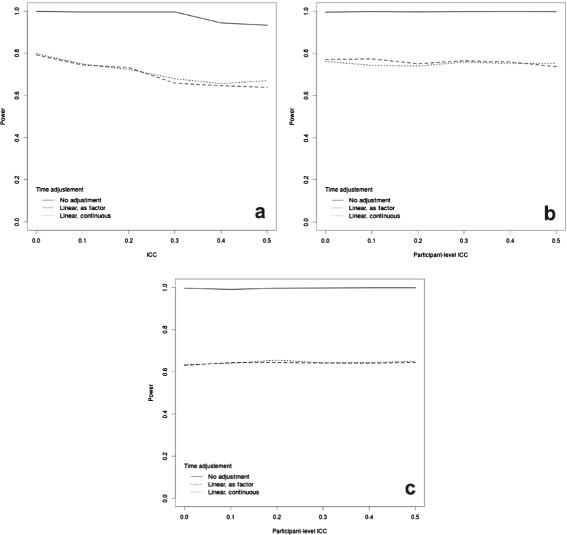
Power curves for a binary outcome assuming 25 clusters, each with 20 subjects and 6 time points at which measurements are taken (including one baseline time). We varied the way in which the assumed linear time effect is included in the model (if at all). Panel (**a**) shows the results for a repeated cohort design; panel (**b**) shows the results for the closed cohort design, assuming a cluster-level ICC of 0.1 and varying the participant-level ICC; panel (**c**) shows the results for the closed cohort design, assuming a cluster-level ICC of 0.5 and varying the participant-level ICC

Figure 6 illustrates the effect on power of misspecification of the time effect in the case of a binary outcome upon varying the assumed values of the ICC. Similarly to what occurs in the continuous outcome case, failure to account for a time effect in the analysis when one is assumed in the DGP results in an overestimation of the power for both repeated cohorts (panel a) and closed cohorts (panels b and c).

Previous research on CRTs has found that modelling time in the analysis substantially reduces the magnitude of the impact of the ICC without reducing the degrees of freedom available for the error term [36]. Given the results of Figs. 5 and 6, this does not appear to be the case for a stepped wedge design, where the impact of varying the ICC is relatively similar for the analysis ignoring and the one including the time effect. We note however that this result may not hold for different specification of the time effect (for example, as a quadratic term).

#### Random intervention effect

We have also evaluated the impact of specifying a model including a random intervention effect. In the simulations, the power decreases considerably upon increasing the assumed standard deviation for the intervention random effect, that is, by assuming increasingly substantial variability in the intervention effect by cluster. For instance, it nearly halves for the binary case described above, when assuming a moderately large standard deviation for the random intervention effect (specifically, a value of *σ*_*u*_=0.3). Of course, as the assumed value for *σ*_*u*_ gets closer to 0, there is less and less difference with the base case, including a fixed intervention effect only. The increase in the underlying variability (and therefore in the resulting sample size) seems to be lower in the case of continuous and normally distributed outcomes.

## Discussion

The claim that SWTs are more efficient than a parallel group CRT in terms of sample size [15] has come under heavy criticism, for example, in [32], where it is suggested that the SWT design is beneficial only in circumstances when the ICC is high, while it produces no advantage as it approaches 0. This finding was corroborated by [37]. Subsequently some of the authors of the original article [15] clarified in a letter [38] that their claims for superior efficiency for the stepped wedge design relate to the option to use fewer clusters, whilst the number of individual participants is often greater. Moreover, HH appear to suggest that the advantage in power from a SWT seen in their work and that of Woertman comes from the increase in the number of participants (assuming as do HH a design with cross-sectional data collected at every crossover) and not the additional randomised crossover points. Kotz et al. [39] argued that power could be amplified to a similar level in standard parallel trials by simply increasing the number of pre- and post-measurements, an assumption supported by Pearson et al. [40], who provided an informal comparison between the implementation of a particular intervention using the stepped wedge design and a non-randomised pre-test-post-test design. This issue has been recently re-examined by Hemming et al. [18], who suggest that a SWT with more than 4 crossover points may be more efficient than a pre-post RCT.

In our work we have also considered the case of cross-sectional data in which each participant provides one measurement to the trial and considered a CRT with the same number of measurements per cluster as a SWT. Under these assumptions, our results are in line with those pointed out above and suggest that, at the cluster size considered, a SWT is more efficient unless the ICC is rather low, for example, much less than 0.1. In other words, given cross-sectional data and the same number of participants measured per cluster, the SWT may often be a more efficient trial design and so will require fewer clusters. The SWT is a design in which a lot of information can be gained from each cluster by increasing the number of measurements per cluster, and is suited to settings where clusters are limited or expensive to recruit. In other settings the costs of adding a cluster to a trial may be low, and it may be more efficient for a given total number of measurements in the trial to conduct a CRT with a large number of clusters (few measurements per cluster) than a SWT with a smaller number of clusters. The CRT would then also be of shorter duration. More generally the costs of a trial may relate to the number of clusters, the trial duration, the total number of participants and the total number of measurements all together in a complex way. Hence, while a SWT is often chosen because there is no alternative trial design, when a SWT or CRT could both be chosen and maximum power is the goal, then the choice between them given the total trial budget requires careful consideration.

In our study, the stepped wedge design was found to be relatively insensitive to variations in the ICC, a finding reported previously in [14]. We also found that in the case where measurements are taken at each discrete time point in the SWT, for a fixed number of clusters the resulting power increases with the number of randomisation crossover points. This is rather intuitive, since for these designs an increase in the number of crossover points equates to an increase in the number of measurements; hence, more information will be available and the number of subjects required will be lower. In practice, the most extreme situation of having one cluster randomised to the intervention at each time point may be unfeasible for these designs. A practical strategy is to simply maximise the number of time intervals given constraints on the number of clusters that can logistically be started at one time point and the desired length of the trial. Moreover, in sensitivity analyses (not shown) it appeared that the gain of increasing the number of crossover points while keeping the number of clusters and the total number of measurements fixed was modest, in comparison with the efficiency gain from adding clusters or measurements to the design. Increasing the number of subjects per cluster may also result in power gains, but as with CRTs, these may be minimal [41].

The failure to consider a time effect when one existed erroneously increased the power. Consequently, we advise researchers to ensure that the effect of time is accounted for in the power calculations, at least as a failsafe measure. Inclusion of time as a factor only minimally reduced the power in comparison to the case in which it was included as a continuous variable, using a linear specification. For generalisability of the time effect and simplicity in the interpretation of the model, it is perhaps even more effective to use a set of dummy variables for the time periods, instead of a single factor [42].

The inclusion of a random intervention effect produced an increase in the resulting sample size; this was an intuitive result, as our simulations assumed an increase in the underlying variability across the clusters. It is worth bearing this possibility in mind when designing a SWT, as the assumption of a constant intervention effect across the clusters being investigated may often be unrealistic, thus leading to potentially underpowered studies. Again, the flexibility of the simulation-based methods allows the incorporation of this feature in a relatively straightforward way.

Not all design possibilities were addressed in our study: for example, the impact of unequal cluster sizes was not considered. In general terms, we would expect a loss of power if the cluster sizes vary substantially, which is consistent with the literature on CRTs [43]. Using a simulation-based approach, relevant information about the expected distribution of cluster sizes in the trial may be easily included in the power computations.

The effect of drop-out was also not fully assessed. This may be relevant, since the extended time required for SWTs may reduce retention, resulting in missing data and loss of power. The impact of drop-out may vary according to how individuals participate in the trial and how measurements are obtained. For cross-sectional data, drop-out can be addressed in a standard manner by inflating the sample size. Drop-out in closed cohort trials, where repeated measurements on individuals are obtained, may be most problematic. Assumptions about the drop-out mechanism and its variation between clusters can be incorporated into a simulation-based approach and their impact on the resulting sample size assessed at the design stage.

Throughout our analysis, time was only considered as a fixed effect. The reason underlying this assumption is that interest was in controlling for temporal trends and fluctuations in prevalence of the outcomes over the course of the particular trials. Including time as a random effect would also result in a more complex model, as adjacent time periods are unlikely to be independent. However, as noted in [14], such an approach might be appropriate if temporal variations in the outcome were thought to be due to factors unrelated to changes in the underlying prevalence of the outcome (such as changes in personnel collecting the outcome data), which may not always be the case.

In line with other articles in this special issue, our work highlights that while SWTs can produce benefits and provide valuable evidence (particularly in implementation research), they are usually also associated with extra complexity in the planning and analysis stage, in comparison to other well-established trial designs. For this reason, it is important to apply the best available methods to carefully plan the data collection. In our work, we have highlighted some of the features that may hinder this process. We plan to make an R package available to allow the practitioners to use both analytical and simulation-based methods to perform sample size calculations in an effective way.

## Conclusions

Our systematic review [11] suggests that, in general, five main methods have been used to calculate sample sizes for SWTs: standard parallel RCT sample size calculations, variance inflation for CRTs, using a specific DE (as in [15]), analytical methods based on normal approximations (such as the method of HH) and simulation-based calculations [24]. Hemming et al. [18] point out that to date no method has been established to compute the required sample size for a SWT under a cohort design.

In general, simulation-based approaches appeared to be a very effective procedure for computing sample size in SWTs, given the constrained nature of DEs and other analytical calculations. For example, complex design features such as varying cluster sizes can be readily incorporated into simulations. Similarly, it is fairly straightforward to investigate differing time effects, that is, linear, exponential or fractional forms. Moreover, currently available analytical forms are based on stepped wedge designs using cross-sectional outcome data measured at discrete time points and thus are not straightforward to adapt to other potential designs. Reliance on sample size calculations for cross-sectional data collection when repeated samples on the same individuals are taken is likely to result in overestimation of the required sample size and thus in wasted resources and unnecessary participation.

## Abbreviations

SWT: Stepped wedge trial
CRT: Cluster randomised trial
RCT: Randomised controlled trial
DE: Design effect
ICC: Intracluster correlation
HH: Hussey and Hughes
CF: Correction factor
DGP: Data generating process
